# Psychometric validation of three new condition-specific questionnaires to assess quality of life, symptoms and treatment satisfaction of patients with aortic aneurysm

**DOI:** 10.1186/s41687-019-0119-0

**Published:** 2019-05-16

**Authors:** Jacquelyn Romaine, George Peach, Matt Thompson, Robert J. Hinchliffe, Clare Bradley

**Affiliations:** 1 Health Psychology Research Unit, Orchard Building, Royal Holloway, University of London, Egham, Surrey, TW20 0EX UK; 2 St George’s Vascular Institute, St George’s Healthcare NHS Trust, London, UK; 3 Endologix Inc and Adjunctive Professor, School of Medicine, Stanford, USA; 4 Bristol Centre for Surgical Research, NIHR Bristol BRC, University of Bristol, Bristol, UK; 5 Health Psychology Research Ltd - Royal Holloway, University of London, Egham, Surrey, TW20 0EX UK

**Keywords:** Abdominal aortic aneurysm, PROMs, Quality of life, Treatment satisfaction, Symptoms, Psychometric development

## Abstract

**Background:**

To evaluate the psychometric properties of three new condition-specific questionnaires designed to assess outcomes amongst patients under pre-operative surveillance for a small abdominal aortic aneurysm (AAA) or who have undergone aneurysm repair. These tools are the Aneurysm-Dependent Quality of Life measure (AneurysmDQoL), the Aneurysm Symptom Rating Questionnaire (AneurysmSRQ) and the Aneurysm Treatment Satisfaction Questionnaire (AneurysmTSQ).

**Results:**

The questionnaires were sent to 297 patients with abdominal aortic aneurysm (AAA) or who had undergone AAA repair (using open or endovascular technique) sampled from five UK NHS Trusts. Exploratory Factor Analysis was used to examine factor structure together with reliability analysis. A subset of 65 patients completed the questionnaires a second time four months later. One hundred and ninety-seven patients (178 men; 18 women) provided data for analysis (69% response rate): mean age was 75 years (range 60–95). Nineteen were under pre-operative surveillance for AAA and 178 had undergone AAA repair (70 open repair; 104 endovascular repair; 4 uncertain). Exploratory Factor Analysis of the AneurysmDQoL and the AneurysmTSQ each demonstrated a one-factor structure. The AneurysmSRQ demonstrated a six-factor structure (emotional, weight loss, lower limb, cognitive, general malaise and gastrointestinal symptoms) and a one-factor composite symptom scale. All scales have clean factor structures: item loadings above 0.40, no cross-loadings, and no factors with fewer than three items. Internal consistency reliability was excellent (α = 0.869–0.959) and test-retest reliability good (Intraclass correlation coefficient = 0.70–0.88).

**Conclusions:**

The three new questionnaires have a clear structure and strong reliability and are now ready for use in clinical trials and routine practice, which will allow evaluation of responsiveness to change.

## Introduction

An abdominal aortic aneurysm (AAA) is a localised dilation of the lower part of the aorta which, if ruptured, is likely to be fatal [1]. Abdominal aortic aneurysms become more common with age and are more common in men [2]. Over time AAAs tend to expand and as they do so, the risk of rupture increases. Small AAAs (3-5 cm) are monitored using periodic ultrasound surveillance and AAA repair is generally only recommended once the aneurysm reaches 5.5 cm - the point where the risk of rupture outweighs the risk associated with elective repair. In 2009, the phased implementation of a national screening program (National Abdominal Aortic Aneurysm Screening Program NAAASP) began in the UK [3]. The NAAASP currently invites all men for ultrasound AAA screening on reaching 65 years of age. Those found to have an AAA are either enrolled into ongoing surveillance or put forward for repair if already at threshold size [4].

Techniques of AAA repair have evolved significantly in recent years with large numbers now treated using endovascular stent-grafts that allow minimally invasive repair. As a result, surgical mortality has fallen dramatically [5] and markers of surgical quality expanded to include patient reported outcomes (PROs), including symptoms, treatment satisfaction and quality of life (QoL). The impact of being made aware of the condition, the need for ongoing surveillance and the need to take new medications (e.g. statins) each have the potential to impact on QoL. Identifying strengths and deficiencies in care from the patients’ perspective can help clinicians strive for even higher quality care rather than simply avoiding morbidity and mortality. The UK Department of Health has, over recent years, undertaken a nationwide initiative to encourage the use of PRO measures (PROMs), both in the surgical specialties in general and in aortic aneurysm surgery specifically. However, until now, no validated aneurysm-specific PROMs exist.

In the absence of a validated aneurysm-specific QoL measure, all previous studies purporting to measure QoL in patients with AAA have used generic tools such as the Medical Outcomes Study Short-Form 36 (SF-36) or the EuroQol-5D (EQ-5D) [6–8]. Although often presented as measures of QoL or ‘health-related quality of life’ (HRQoL), these tools are actually measures of health status (i.e. physical and mental function) rather than true QoL. Quality of life is a much broader concept that incorporates (but is not limited to) how dysfunctional physical and/or mental status and other demands of a condition and its treatment may impact upon patients’ lives. To assess the impact of AAA and its treatment on QoL, three new condition-specific questionnaires have been developed using an iterative process of focus groups and in-depth interviews involving patients with AAA [9]. The newly designed questionnaires are: The Aneurysm-Dependent Quality of Life Questionnaire (AneurysmDQoL), the Aneurysm Symptom Rating Questionnaire (AneurysmSRQ) and the Aneurysm Treatment Satisfaction Questionnaire (AneurysmTSQ).

### The AneurysmDQoL

**Fig. 1:**
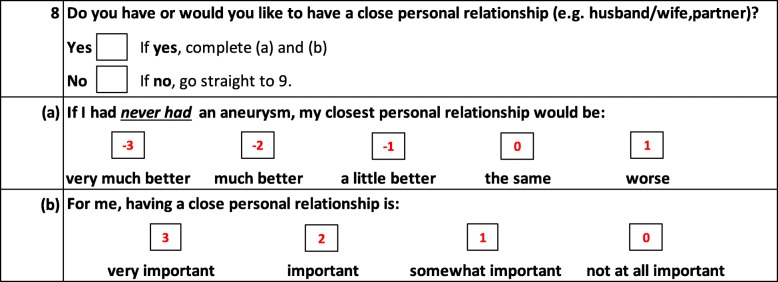
Example item from the AneurysmDQoL (numbers indicate score assigned to each rating)

The design of the AneurysmDQoL was based upon the Audit of Diabetes-Dependent Quality of Life (ADDQoL) - a widely used questionnaire designed for use by people with diabetes [10, 11]. The ADDQoL has also been adapted for use by people with many other conditions including renal disease (RDQoL), macular disease (MacDQoL), growth hormone deficiency (HDQoL), hypothyroidism (ThyDQoL) and diabetic retinopathy (RetDQoL) [12–16]. Influenced by the way QoL was conceptualized in the design of the SEIQoL (Schedule for the Evaluation of Individual Quality of Life) interview methodology [17], the AneurysmDQoL recognizes individual differences in the experience of quality of life. Unlike the majority of QoL and health status tools, respondents indicate if an aspect of life (e.g. work) is not relevant to them and, for those aspects that are of relevance, they are asked to rate not only how much each aspect of their life has been affected by their condition (impact), but also how important they consider this aspect of their life to be for their QoL (importance) (Fig. 1).

Part (a) of each item is scored from − 3 (greatest negative impact) to + 1 (positive impact) and part (b) is scored from 3 to 0 (very important to not at all important). The ‘Weighted Impact’ (WI) of AAA on that particular aspect of life is then calculated by multiplying the impact score by the importance score (scores range from − 9 to + 3). In this way the AneurysmDQoL is sensitive to the fact that any given aspect of life may have different significance to different individuals and therefore is likely to have varying impact on QoL, and that the importance of a particular aspect of life may change over time even for the same individual. An Average Weighted Impact score (AWI), can be obtained by summing the WI scores and dividing by the number of applicable domains (scores range from − 9 to + 3). Thus, scoring ignores non-applicable domains and gives greater emphasis to domains of greater importance to the individual, providing a highly personalised assessment of the impact of AAA on an individual’s QoL.

The AneurysmDQoL includes 24 questions in total: two initial overview items - current QoL and how QoL would be different if respondents had not had an aneurysm, and 22 domain-specific items (e.g. family, household tasks) [9].

### The AneurysmSRQ

The AneurysmSRQ is a measure of symptoms associated with AAA and its treatment which assesses the degree to which patients are bothered by applicable symptoms. The format of the questionnaire was developed in earlier work with patients who had hypothyroidism [18] and has since been adapted for other chronic conditions including hypoglycaemia [19]. The AneurysmSRQ comprises 44 items, each divided into two parts. Part (a) asks respondents to indicate if they have experienced the symptom in recent weeks, regardless of the cause. Part (b) asks respondents to indicate how much the symptom bothers them. If the answer to part (a) is *‘No’*, respondents are asked to go straight to the next symptom. Applicable symptoms are scored on a scale from 1 (not at all) to 4 (a lot).

### The AneurysmTSQ

The Aneurysm Treatment Satisfaction Questionnaire (AneurysmTSQ) is designed to measure treatment satisfaction in people living with AAA. Based on the format of the Diabetes Treatment Satisfaction Questionnaire [20] and other -TSQ measures [21–23] the AneurysmTSQ explores multiple aspects of treatment satisfaction (e.g. side-effects, convenience). The AneurysmTSQ includes 11 Likert scale items which are rated from 6 (very satisfied) to 0 (very dissatisfied). Items 1 to 7 are designed for patients undergoing surveillance of small AAAs as well as those who have had aneurysm repair (e.g. information, staff support). Items 8 to 11 are designed to assess post-operative aspects of the treatment process (e.g. side-effects, follow-up) and are therefore only suitable for patients following aneurysm repair. Items can be used individually or they can be summed to give a total treatment satisfaction score.

The present paper focuses on the psychometric validation of the new tools, involving examination of their overall structure (including any subscales), internal consistency and test-retest reliability. It reports optimal scoring methods following use in clinical practice or research.

## Methods

### Design

A cross-sectional survey design was used, with patients at various points in the treatment pathway asked to complete the three new questionnaires. At the time of data collection, the national screening program had yet to be fully rolled out, however in anticipation of this, all individuals diagnosed with an AAA were invited to take part. The time-points chosen for completion were: pre-operative intervention (surveillance of AAAs < 5.5 cm) and 6 weeks, 3 months, 6 months, 12 months and > 12 months post-repair.

### Participants

Questionnaires were completed by 297 patients diagnosed with AAA purposively sampled from five UK NHS Trusts. Respondents included those under surveillance for a small AAA and those who had undergone an AAA repair. Target sample size was based on a participant to variable ratio of 4:1 (minimum 100). Previous research using –DQoL and –TSQ measures for other conditions have demonstrated strong reliabilities, high factor loadings and a clean structure with such sample sizes e.g. [22–24]. Data from 197 patients were available for analysis (69% response rate).

The majority of participants were male. The mean age for women was slightly higher than for men and a higher number of men than women currently, or previously, smoked: men = 72%, women = 45% (Table 1). The majority of both men and women reported elective EVAR as their AAA treatment (elective: men = 68%, women = 72%; EVAR: men = 51%, women = 78%). Mean time between operation and completing the questionnaire was slightly higher for men than women (Table 2).

**Table 1:** Participant gender, age and smoking status

Sex		Age						Smoker				
	N	Mean	SD	Min	Max	N	Missing	Yes	No	Ex-smoker	N	Missing
Male	178	74.78	7.74	60	94	154	24	17	50	111	177	0
Female	18	77.24	9.17	64	95	12	6	3	10	5	18	0
Total	196	74.96	7.85	60	95	166	30	60	20	116	195	0

**Table 2:** Descriptive statistics by gender for treatment course, operation type and time since operation

Treatment Course						Operation Type				Time Since Operation (Months)			
*Sex*	Pre-Op	Elective	Rupture	N	Missing	Open	EVAR	N	Missing	Mean	SD	N	Missing
Male	16	121	10	178	31	68	90	158	20	12.13	6.78	126	52
Female	2	13	0	18	3	2	14	16	2	10.08	5.51	12	6
Total	18	132	10	196	34	70	104	174	22	11.96	6.69	138	58

Ethnicity of participants was predominantly White (men = 96%, women = 100%). A subset of patients (*n* = 65) were asked to complete the questionnaire pack a second time (mean duration to retest = 4 months; range 1–7 months) to allow assessment of test-retest reliability.

### Procedure

Prior to the start of the study the UK National Research Ethics Service (NRES Committee – London Chelsea − 11/LO/1416) approved the process of patient recruitment and patients provided informed written consent at each stage. Patients were recruited from five NHS Healthcare Trusts: St George’s Healthcare NHS Trust, North Bristol NHS Trust, Worcestershire Acute Hospitals NHS Trust, Norfolk and Norwich University Hospitals NHS Trust and University Hospital Southampton NHS Foundation Trust. Other than St George’s Hospital (chosen for the large numbers of EVAR aneurysm repairs performed), these centres were purposefully selected for this study as they carry out significant numbers of both OR and EVAR procedures. In each centre, members of the healthcare team contacted all patients who had undergone aneurysm repair within the preceding 12 months and invited them to participate in the study. Southampton also invited patients who had undergone aneurysm repair between 12 and 24 months prior to the study. Two centres identified a number of patients enrolled in preoperative surveillance of small AAAs. Patients who expressed interest were then sent the questionnaire pack (stamped addressed envelope included for return). The pack included an information sheet, consent form, detailed instructions and the questionnaires.

### Analytic approach

Prior to conducting the analyses, the data were screened and relevant assumptions assessed. The correlation matrix was inspected for poorly correlated items (*r* < 0.3) and MSA (Measure of Sampling Adequacy) coefficients for each variable checked (< 0.6 problematic). The Kaiser-Meyer-Olkin value was checked to ensure it exceeded the recommended value of 0.6 [25, 26] and Bartlett’s Test of Sphericity [27] examined for statistical significance.

Exploratory Factor Analysis (EFA) was conducted in two stages. Initial analyses were carried out using a principal components analysis (PCA: data reduction retaining as much information as possible). Components were considered for retention using three decision rules: Kaiser’s criterion (eigenvalues > 1), inspection of the Scree plot and Horn’s parallel analysis [28]. Once the components had been identified, a fixed factor EFA was run (examining data structure and underlying construct(s) using shared variance). All initial analyses were run using an oblique (Direct Oblimin) rotation, to allow for potential inter-correlations between components. Factors were considered to be related if they had a correlation coefficient > 0.32. Due to the non-normal distribution of the data, Principal Axis Factoring (PAF) was the chosen method of extraction [29, 30]. Examination of the factor loadings focused on identifying the ‘cleanest’ factor structure (item loadings above 0.40 [31], no or few item cross-loadings [> 0.32], and no factors with fewer than three items) [27]. Factor loading strength was guided by: fair (0.45), good (0.55), very good (0.63), excellent (0.71) [32]. Poorly performing items were dropped from the analysis one at a time and the analysis rerun. All analyses used listwise deletion of cases.

Internal consistency reliability analysis was run to determine how many missing responses can be tolerated when calculating a total scale score [33]. The strongest contributing scale item was dropped and the analysis rerun until alpha < 0.7 or 50% of items had been dropped. Intraclass correlation coefficients (ICC) were used to assess test-retest reliability (two-way random, single, absolute). Intraclass correlation coefficients were guided by: excellent (≥ 0.81), good (0.61–0.80), moderate (0.41–0.60), poor (≤ 0.40).

## Results

### Exploratory factor analysis of the AneurysmDQoL

A complete set of responses from 157 participants (16 under surveillance; 141 post-repair) were included in the analysis of the AneurysmDQoL. Examination of data suitability led to the removal of Item 2: work. Reasons for this included an MSA value of 0.349 and 160 out of 197 participants reporting work as non-applicable).

Principal components analysis suggested a one-factor solution. The forced one-factor EFA found that the 22 items in the AneurysmDQoL accounted for 51.99% of the total variance within the data. Analysis of the factor matrix however revealed Item 15 (finance) loaded at 0.350. After removal of Item 15 (finance) the lowest loading item was Item 23 (value each day). The relatively weak loading (0.472) combined with problems highlighted during the design phase (meaning unclear) led to the removal of Item 23. The rerun forced one-factor 20-item EFA now explained 55.54% of the variance in the data. All items now loaded > 0.5. Cronbach’s-α coefficient of internal consistency was very high and test-retest reliability was good. Internal consistency reliability run to determine how many missing responses can be tolerated when calculating a total scale score revealed that the AneurysmDQoL remains reliable (α > 0.85) even if patients omit responses for up to eight core items (items with no non-applicable option). Table 3 demonstrates factor loadings, communalities and reliability coefficients.

**Table 3:** AneurysmDQoL - Factor loadings, communalities and reliability coefficients (pre and post-repair patients), ordered by factor loadings

Quality of life domain	Factor 1	Communalities	Alpha if item deleted (Original alpha = 0.959)
Household tasks	0.865	0.748	0.956
Family life	0.864	0.746	0.956
Friendships	0.837	0.701	0.956
Holiday	0.836	0.698	0.956
Getting out & about	0.831	0.691	0.956
Energy	0.829	0.687	0.956
Physical	0.819	0.671	0.956
Leisure	0.796	0.634	0.957
Do things for others	0.792	0.628	0.957
Health	0.791	0.626	0.957
Feelings about the future	0.774	0.599	0.957
Long journeys	0.768	0.590	0.957
Physical discomfort	0.747	0.557	0.957
Anxiety	0.688	0.473	0.958
Depend on others	0.687	0.471	0.958
Personal relationship	0.659	0.434	0.958
Others worry about me	0.583	0.340	0.959
Think clearly	0.546	0.299	0.960
Enjoy food	0.510	0.230	0.960
Sex life	0.506	0.256	0.960
Percentage total variance = 55.54	Number of items in scale = 20	Alpha = 0.959	ICC = 0.70

To ensure the tool would be suitable for use by pre- and post-repair patients, analyses were rerun with post-repair patients only (*n* = 141). A 20-item single-factor solution again revealed the cleanest structure, explaining 55.38% of the variance. All items loaded > 0.48 and Cronbach’s-α coefficient of internal consistency was again high (0.959) and test-retest reliability good (0.66). The results of these analyses support the use of a single-scale, 20-item AneurysmDQoL as a measure of the impact of AAA on quality of life for patients under surveillance for a small AAA and for patients following AAA repair.

### Exploratory factor analysis of the AneurysmSRQ

Responses from 164 participants were included. Low MSA values led to the removal of three items (pain in buttocks 0.478, wound infection 0.572, difficulty urinating 0.568). Principal components analysis of the remaining 41 items (*n* = 166) revealed a six-component solution. A forced-six factor EFA (oblique rotation) explained 42.38% of the total variance within the data. Examination of the factor loadings led to the stepwise removal of 17 items.

The 24-item six-factor solution accounted for 52.62% of the variance within the data and all items loaded > 0.43. All factors demonstrated good internal consistency and moderate to excellent test-retest reliability. Details of factor names, factor loadings, communalities, and reliability coefficients are presented in Table 4.

**Table 4:** AneurysmSRQ Forced six-factor EFA - factor loadings, communalities and reliability coefficients

Symptom	Factor 1: Emotion	Factor 2: Appetite	Factor 3: Lower Limb	Factor 4: Cognitive	Factor 5: Malaise	Factor 6: Gastro	Communalities	Subscale alpha if item deleted
Emotional	***0.817***	0.167	0.025	−0.032	0.011	− 0.076	0.675	0.834
Panic	***0.746***	−0.183	−0.103	− 0.100	0.024	0.132	0.586	0.865
Angry	***0.724***	−0.055	0.140	0.076	0.005	0.040	0.667	0.847
Depressed	***0.668***	0.148	0.027	0.084	0.051	0.160	0.689	0.850
Worried	***0.663***	0.070	0.069	0.183	0.012	−0.049	0.570	0.851
Lost weight	−0.096	***0.783***	0.069	0.013	0.113	0.081	0.696	0.630
Poor appetite	0.150	***0.650***	0.010	0.000	−0.009	0.023	0.466	0.536
Nausea vomited	0.017	***0.487***	−0.025	−0.070	0.299	0.140	0.463	0.715
Pain in calves	−0.048	0.078	***0.731***	0.009	−0.014	0.026	0.535	0.709
Tingling legs/feet	0.005	0.182	***0.716***	−0.022	−0.107	0.062	0.537	0.712
Heaviness in legs	0.074	−0.159	***0.639***	0.045	0.081	0.121	0.578	0.679
Weakness in legs	0.025	−0.020	***0.445***	0.193	0.189	−0.006	0.377	0.735
Swollen legs	0.145	−0.223	***0.433***	−0.035	0.153	−0.008	0.322	0.751
Memory problems	−0.051	−0.085	− 0.130	***0.896***	0.034	0.075	0.777	0.726
Difficulty thinking	0.035	0.105	0.126	***0.820***	−0.070	−0.090	0.721	0.760
Difficulty concentrating	0.314	−0.058	0.130	***0.518***	−0.012	0.117	0.610	0.836
Minor Illnesses	−0.029	0.018	0.105	−0.052	***0.713***	−0.057	0.515	0.580
Diarrhoea	−0.045	0.012	−0.033	0.005	***0.633***	0.079	0.417	0.612
Feverish	0.090	0.174	−0.029	−0.035	***0.562***	−0.062	0.394	0.628
Headaches	0.094	−0.082	0.038	0.130	***0.456***	0.131	0.356	0.659
Bloated	−0.066	−0.021	0.178	−0.024	− 0.082	***0.637***	0.429	0.653
Abdominal pain	0.205	0.090	−0.056	0.111	0.060	***0.550***	0.517	0.650
Flatulence	−0.043	0.027	0.012	0.142	0.158	***0.549***	0.431	0.672
Indigestion	0.091	0.067	−0.034	−0.067	0.003	***0.512***	0.301	0.692
Percentage total variance	24.99	8.89	6.56	4.87	3.93	3.46		
Items in scale	5	3	5	3	4	4		
Alpha	0.88	0.72	0.76	0.84	0.69	0.73		
ICC	0.69	0.47	0.69	0.82	0.59	0.80		

Strongest factor loadings in bold

To assess the importance of each item and clarify whether items not included in the six subscales should be removed from the questionnaire, the frequency and bother ratings of each item were examined (Table 5 and 6). This process demonstrated that while similar patterns of response were not found among these items (therefore not included in the subscales), many patients experienced these symptoms and/or described them as causing moderate or severe bother. It was therefore decided that these items would remain in the questionnaire as stand-alone items. Frequency responses also demonstrate no floor or ceiling effects (> 15% having highest / lowest score) [34].

**Table 5:** AneurysmSRQ frequency scores and bother ratings for all items included in the six subscales, ordered by item number

Symptom	Mean	SD	No experience of symptom	Bother Rating				Total N	Bothered by symptom %
				Not at all	A Little	Moderate	A lot		
Headaches	2.54	0.79	167	2	12	11	3	195	14
Feverish	2.58	1.00	180	2	3	5	2	192	6
Pain/discomfort in calves	2.66	0.77	125	1	34	24	12	196	36
Abdominal pain	2.76	0.79	155	0	17	13	8	193	20
Minor Illnesses	2.38	0.95	143	10	19	16	7	195	27
Depressed or low	2.75	0.81	141	1	22	19	11	194	27
Feelings of panic	3.00	0.52	175	0	2	12	2	191	8
Worried/nervous	2.57	0.69	147	1	22	19	4	193	24
Irritable/angry	2.76	0.64	156	0	13	20	4	193	19
Emotional/upset	2.74	0.73	152	1	15	20	6	194	22
Difficulty concentrating	2.68	0.76	146	1	20	19	7	193	24
Memory problems	2.45	0.80	128	5	36	20	8	197	35
Difficulty thinking	2.42	0.71	140	3	30	18	4	195	28
Tingling legs/feet	2.54	0.70	127	1	37	24	7	196	35
Heaviness in legs	2.57	0.79	141	1	30	14	9	195	28
Weakness in legs	2.77	0.70	128	0	25	31	10	194	34
Swollen legs	2.48	0.75	168	2	12	11	2	195	14
Poor appetite	2.59	1.10	174	4	7	5	6	196	11
Lost weight	2.19	1.05	159	11	14	6	6	196	19
Indigestion or heartburn	2.41	0.79	147	4	26	14	5	196	25
Nausea/vomited	2.71	0.85	179	1	6	7	3	196	9
Flatulence or belching	2.63	0.88	124	5	31	22	14	196	37
Bloated	2.68	0.75	165	1	12	14	4	196	16
Diarrhoea	2.75	0.70	168	0	11	13	4	196	14

Mean scores exclude participants not experiencing a particular symptom

**Table 6:** AneurysmSRQ frequency scores and bother ratings for all stand-alone items ordered by item number

Symptom	Mean	SD	No experience of symptom	Bother Rating				Total N	Bothered by symptom %
				Not at all	A Little	Moderate	A Lot		
Tired	2.76	0.78	103	4	29	43	15	194	47
Pain/discomfort thighs	2.62	0.80	142	2	24	18	8	194	27
Pain/discomfort groin	2.71	0.80	159	0	19	11	8	197	19
Pain/discomfort buttocks	2.85	0.77	170	0	10	11	6	197	14
Pain/discomfort back	2.86	0.79	113	1	30	33	20	197	43
Wound Infection	2.56	1.01	186	1	4	2	2	195	5
Excessive bruising	2.30	0.87	174	4	7	8	1	194	10
Unsteady/uncoordinated	2.71	0.84	135	2	27	20	13	197	31
Dizzy/lightheaded	2.52	0.82	152	1	27	8	8	196	22
Trembling e.g. limbs	2.47	0.64	180	0	9	5	1	195	8
Lost interest in sex	2.60	0.96	145	7	14	18	9	193	25
Avoid sexual activity	2.67	0.77	178	1	6	9	2	196	9
Problems sexual function	2.83	0.95	136	6	14	23	16	195	30
Excessive sweating	2.72	0.94	170	2	9	8	6	195	13
Episodes too hot/cold	2.64	0.78	135	4	21	29	7	196	31
Sleep problems	2.84	0.81	134	3	17	29	13	196	32
Generally weak	2.67	0.81	136	3	22	27	8	196	31
Gained weight	2.03	0.77	158	9	18	8	1	194	19
Constipation	2.66	0.94	152	4	15	13	9	193	21
Difficulty urinating	2.44	0.71	167	1	14	8	2	192	13

Mean scores exclude participants not experiencing a particular symptom

Though six clear subscales had been identified within the AneurysmSRQ, the potential pragmatic benefits of having a single summable symptom scale were also recognised. In order to identify the maximum number of items that could be combined into a single scale a forced one-factor EFA was run. Examination of the factor loadings led to the removal of 17 low-loading items. The final one-factor solution comprised 24 items. All items loaded > 0.4, and explained 30.15% of variance in the data. This factor was titled the ‘Composite Symptom Scale’ and provides the broadest single overall score for bother from symptoms. Internal consistency reliability was very high and ICC excellent.

Internal consistency reliability analysis run to determine how many missing responses can be tolerated when calculating the AneurysmSRQ Composite Symptom Scale revealed that the scale remains reliable even if patients omit responses for up to 12 items. Table 7 demonstrates factor loadings, communalities and reliability coefficients.

**Table 7:** AneurysmSRQ composite symptom score - factor loadings, communalities and reliability coefficients, ordered by factor loading

Symptom	Factor Loading	Communalities	Alpha if item deleted (Overall alpha = 0.906)
Depressed or low	0.764	0.583	0.898
Generally weak	0.727	0.528	0.898
Tired or lethargic	0.702	0.493	0.898
Irritable or angry	0.683	0.466	0.900
Difficulty concentrating	0.670	0.449	0.899
Worried or nervous	0.647	0.419	0.901
Emotional or upset	0.602	0.363	0.901
Abdominal pain	0.577	0.333	0.902
Episodes too hot or cold	0.539	0.290	0.902
Feelings of panic	0.535	0.286	0.903
Pain or discomfort in back	0.520	0.270	0.903
Weakness in legs	0.518	0.268	0.902
Trembling e.g. limbs	0.516	0.266	0.904
Pain or discomfort in groin	0.482	0.233	0.903
Unsteady or uncoordinated	0.475	0.225	0.903
Headaches	0.467	0.218	0.903
Heaviness in legs	0.460	0.212	0.903
Excessive sweating	0.459	0.211	0.904
Flatulence or belching	0.454	0.207	0.904
Memory problems	0.439	0.193	0.904
Difficulty thinking	0.439	0.192	0.904
Dizzy or lightheaded	0.435	0.190	0.904
Avoided sexual activity	0.422	0.178	0.905
Feverish	0.403	0.163	0.905
Percentage total variance = 30.15		Number of items in scale = 24	ICC = 0.81

### Exploratory factor analysis of the AneurysmTSQ

The AneurysmTSQ includes 11 items, all of which are suitable for patients who have undergone AAA repair. Items one to seven are also suitable for patients undergoing AAA pre-repair surveillance.

#### Post-repair patients

Treatment satisfaction data were obtained from 154 post-repair patients. Data suitability checks all proved satisfactory. A PCA of the 11 AneurysmTSQ items suggested a one component solution. The forced-one-factor EFA explained a total of 49.69% of the total variance within the data. Examination of the factor loadings revealed all items loaded > 0.476. Cronbach’s alpha was strong and ICC demonstrated excellent test-retest reliability. Details of the factor loadings, communalities and reliability coefficients are presented in Table 8. These results support the use of the 11-item AneurysmTSQ as a measure of total treatment satisfaction for patients who have received post-operative care for an aortic aneurysm.

**Table 8:** AneurysmTSQ factor loadings, communalities and reliability coefficients: Post-repair patients

Aspect of Treatment	Factor Loading	Communalities	Alpha if Item Deleted (Overall alpha = 0.902)
Treatment	0.843	0.711	0.886
Information	0.831	0.690	0.892
Post-op follow-up	0.829	0.687	0.884
Support staff	0.826	0.683	0.886
Convenience	0.724	0.525	0.891
Understand treatment	0.713	0.509	0.892
Stay length	0.666	0.443	0.893
Results feedback	0.631	0.398	0.898
Encourage others	0.571	0.326	0.898
Discomfort or pain	0.518	0.268	0.901
Side-effects	0.476	0.227	0.906
Percentage total variance = 49.69		Number of items in scale = 11	ICC = 0.88

#### Pre and post-repair patients

Treatment satisfaction data were obtained from 182 participants. Consistent with the 11-item EFA, PCA using items 1–7 revealed a one-factor solution. The seven-item one-factor solution accounted for 52.83% of the variance within the data. Cronbach’s alpha coefficient was strong and test retest reliability was good. Factor loadings, communalities and reliability coefficients are demonstrated in Table 9.

**Table 9:** AneurysmTSQ factor loadings, communalities and reliability coefficients: Pre and post-repair patients

Aspect of Treatment	Factor Loading	Communalities	Alpha if Item Deleted (Overall alpha = 0.869)
Support staff	0.830	0.689	0.833
Treatment	0.826	0.682	0.839
Information	0.793	0.628	0.838
Understand treatment	0.771	0.594	0.841
Convenience	0.678	0.459	0.853
Results feedback	0.678	0.459	0.858
Discomfort or pain	0.432	0.186	0.888
Percentage total variance = 52.83		Number of items in scale = 7	ICC = 0.78

These results demonstrate support for a separate 7-item subscale suitable for use by patients pre-repair surveillance in addition to the full 11-item measure of treatment satisfaction for patients who have received post-operative care for AAA.

## Discussion

The psychometric analyses presented here provide detailed information on the structure of the AneurysmDQoL, AneurysmSRQ and AneurysmTSQ and strongly support their validity for use by patients with AAA.

The content validity of the three new tools was established through an iterative design process involving patients at every stage to ensure all included items were relevant to this patient group and no potentially important items were missing [9]. Strong evidence of the construct validity of each tool has been demonstrated here through psychometric analysis. All three tools have a clear structure, strong internal consistency and good test-retest reliability.

During the validation process, the value of single factor solutions was recognised as they have pragmatic advantages for clinical use. The data supported single factor solutions for both the AneurysmDQoL and the AneurysmTSQ. Analysis of the AneurysmDQoL confirmed that 20 of the initial 23 items could be combined into a single scale with the item ‘value each day’ requiring removal and the work and finance items being retained in the questionnaire but not included in the scale. It is perhaps not surprising that these particular items stood apart, since the majority of patients undergoing AAA repair have retired from work. Nonetheless, for those who *are* still in employment, the impact on work and finances could potentially be profound and the decision was therefore taken to retain these two items in the questionnaire as stand-alone items. In the UK, AAA medical care is provided free at the point of use. In countries where this is not the case, finances may be more impacted by AAA. Given that finance loaded only marginally lower than the 0.4 cut off and made a negligible difference to the internal consistency of the scale this item could, if needed, be included in computing AWI in countries where medical care is not provided free of charge or in multinational clinical trials where finances may be more relevant in some countries.

The poor performance of the ‘value each day’ item was not unexpected as several patients had found the item difficult to understand during pilot testing. This item was therefore completely removed from the questionnaire, resulting in the final 22-item version of the AneurysmDQoL with 20 items contributing to the scale score.

No changes were made to the AneurysmTSQ following psychometric analysis. All items loaded onto a single factor with good reliability. The data also suggest that the first seven items can be used as a separate subscale for patients who are currently under surveillance following diagnosis of a small AAA but have not undergone repair. Due to the small numbers of patients in the dataset currently under surveillance, analysis of this subscale was conducted using data from both pre and post-repair individuals who had undergone surveillance. Although the patient-centred design of the measure provides strong content validity [9] and the free text box provided with each questionnaire (allowing respondents to report for example, any aspects of treatment satisfaction not covered in the questionnaire) suggested no further additions were needed, further work with a larger pre-repair surveillance dataset is required for confirmation of the psychometric properties of the questionnaire when used only with patients undergoing surveillance for small aneurysm.

In contrast to the other tools, analyses demonstrated that the 44 items in the AneurysmSRQ could not be combined into a single scale that included all items. However, stepwise removal of items not contributing to a single scale demonstrated that it was possible to combine a total of 24 items into a single ‘Composite Symptom Score’. Though this may not be a truly comprehensive score, it does provide the broadest possible single indicator of symptom burden for these patients. Psychometric analysis also showed that six subscales could be identified with the AneurysmSRQ: emotion; appetite; lower-limb symptoms; cognitive function; general malaise; and gastrointestinal symptoms. These subscales comprised a total of 24 items (not identical to composite symptom score items). Importantly, although 20 items had to be excluded from the subscales on the basis of the psychometric analysis, data from focus groups and patient interviews had suggested that a number of these excluded items were important to patients. Indeed, some items may have failed to load onto subscales not because they are irrelevant or anomalous, but because they are more general in nature. For example, tiredness and back pain were each experienced by more than 40% of respondents (Table 6). Furthermore, some of the non-scale items with very low frequencies and bother ratings (e.g. wound infection; bruising) are most likely to be experienced by patients in the early post-repair group – a group relatively under-represented in this study. The decision was therefore taken to retain all non-scale items in the questionnaire until further data are gathered to justify more fully their retention or exclusion.

In addition to consistency and reliability, the criterion validity of new questionnaires can also be evaluated. Criterion validity is an assessment of the performance of a new tool relative to a known ‘gold-standard’ measure. In developing new QoL measures, however, this can be problematic since there is often no existing gold standard. This is the case for patients with AAA. The systematic literature review performed prior to questionnaire development demonstrated that almost all previous attempts to describe QoL amongst patients with AAA have actually used health status measures (such as the SF-36 and EQ-5D) and have yielded an array of conflicting results [7]. The disagreement in previous results suggested that those tools may not have been suitable for purpose and prompted the development of the new questionnaires. It therefore follows that those tools cannot be used as the standard against which the new questionnaires are measured. Though comparison to a ‘gold standard’ was not possible, the need for such external validation was minimised by the fact that the format of the –DQoL, −TSQ and –SRQ measures have been tried and tested for many other patient groups and many of the items in the new questionnaires were derived from item libraries created in the course of designing and developing the measures for other patient population tools [12, 13, 18–20, 22, 24, 35–40].

The data collected in this study were sufficient for overall psychometric analysis (based on participant number, clean structure and number of high loading items) and indicate that the new tools are valid for both pre-repair surveillance and post-repair patients. Nonetheless, a larger dataset that included a greater number of pre-repair surveillance patients would have allowed for direct confirmation of the psychometric properties for pre-repair patients as a separate group. It is also noted that the male: female distribution in our study population was 9:1 whilst national data on the prevalence of the condition shows the male female ratio to be 6:1 [41]. It is unclear whether this ratio is truly representative of patients undergoing aneurysm repair or whether women were less often invited or less inclined to take part in the study. However, a similar or greater proportion of women patients was also observed in some of the largest studies of AAA repair to date, including the EVAR-1 (9%), EVAR-2 (15%) and UKSAT (18%) trials [42–44]. In addition, whilst the initial intention had been to include a longitudinal cohort of patients, this was ultimately not possible and for logistical reasons we conducted a cross-sectional study of patients at different stages pre- and post-aneurysm repair. Thus we have been able to examine subgroup differences [45] but have yet to assess the tools’ responsiveness to change. This will need to be examined in future research.

## Conclusions

The increasing number of patients now attending screening and undergoing EVAR repair, with its associated follow-up and increased risk of further intervention, suggests an urgent need for condition-specific AAA PROs measures. The AneurysmDQoL, AneurysmSRQ and AneurysmTSQ are three new condition-specific questionnaires that will allow clinicians to assess quality of life, impact of symptoms and treatment satisfaction of patients with AAA before or after repair. We have published elsewhere evidence of their acceptability to patients [9] and evidence of between group differences [45] and here reported clear structure, good internal consistency, and test-retest reliability. Responsiveness to change now needs to be assessed.

## Abbreviations

AAA: Abdominal Aortic Aneurysm
ADDQoL: Audit of Diabetes-Dependent Quality of Life
AneurysmDQoL: Aneurysm Dependent Quality of Life
AneurysmSRQ: Aneurysm Symptom Rating Questionnaire
AneurysmTSQ: Aneurysm Treatment Satisfaction Questionnaire
AWI: Average Weighted Impact
EFA Exploratory: Factor Analysis
EQ-5D: EuroQoL-5 Dimensions
EVAR: Endovascular Aortic Repair
HDQoL: Hormone Deficiency-Dependent Quality of Life
ICC: Intraclass Correlation Coefficient
MacDQoL: Macular Disease Dependent Quality of Life
MSA: Measure of Sampling Adequacy
NAAASP: National Abdominal Aortic Aneurysm Screening Programme
NRES: National Research Ethics Service
OR: Open Repair
PAF: Principal Axis Factoring
PCA: Principal Components Analysis
PROMs: Patient Reported Outcome Measures
QoL: Quality of Life
RDQoL: Renal-Dependent Quality of Life
RetDQoL: Retinopathy-Dependent Quality of Life
SEIQoL: Schedule for the Evaluation of Individual Quality of Life
SF-36: (Medical Outcomes Study) Short-Form 36
ThyDQoL: (Underactive) Thyroid-Dependent Quality of Life
WI: Weighted Impact
